# Microscopic Particles in Two Fractions of Fresh Cerebrospinal Fluid in Twins with Schizophrenia or Bipolar Disorder and in Healthy Controls

**DOI:** 10.1371/journal.pone.0045994

**Published:** 2012-09-25

**Authors:** Viktoria Johansson, Rolf Nybom, Lennart Wetterberg, Christina M. Hultman, Tyrone D. Cannon, Anette G. M. Johansson, Carl Johan Ekman, Mikael Landén

**Affiliations:** 1 Department of Medical Epidemiology and Biostatistics, Karolinska Institutet, Stockholm, Sweden; 2 Department of Neuroscience, Karolinska Institutet, Stockholm, Sweden; 3 Department of Clinical Neuroscience at St. Göran, Karolinska Institutet, Stockholm, Sweden; 4 Departments of Psychology and Psychiatry and Biobehavioral Sciences, University of California Los Angeles, Los Angeles, California, United States of America; 5 Institute of Neuroscience and Physiology, The Sahlgrenska Academy at Gothenburg University, Gothenburg, Sweden; University of North Dakota, United States of America

## Abstract

**Background:**

Using scanning electron microscopy, microscopic structures have been identified in fresh cerebrospinal fluid (CSF) in patients with schizophrenia and bipolar disorder, but only rarely in control subjects. However, it has not been determined whether these microscopic particles represent state or trait markers, i.e. if their presence is related to clinical manifestations of the disease or if they also can be found in as yet asymptomatic individuals with a genetic liability. This question can be addressed by studying twins discordant or concordant for schizophrenia or bipolar disorder.

**Methodology/Principal Findings:**

We investigated microscopic structures in CSF in 102 individuals: 21 monozygotic and 16 dizygotic twins affected or not affected with schizophrenia, schizoaffective disorder or bipolar disorder and in 65 healthy singleton controls. A first and a second fraction of CSF was freshly applied on filters and examined by scanning electron microscopy technique. Spherical particles with lipid appearance averaging between 0.1 to 8.0 µm in diameter were detected in the center of the filter as well as located in the margins of larger aggregates binding in a viscous state. Structures were found in 12 of 17 probands, 5 of 12 healthy co-twins and 3 of 73 healthy controls. Thus, a positive microscopic finding significantly increased the likelihood of belonging to the proband group (OR = 48, 95% CL: 8.2–550, p<0.0001) and the co-twin-group (OR = 16, 95% CL: 2.0–218, p = 0.006). Age, sex, history of alcohol abuse or anxiety syndrome, somatic disorder and markers of acute inflammatory activity did not account for group differences; nor did exposure to psychotropic medication.

**Conclusion:**

Presence of microscopic particles in CSF may possibly reflect trait dependent genetic or environmental vulnerability in patients with schizophrenia, schizoaffective disorder or bipolar disorder.

## Introduction

Identifying biomarkers for psychiatric disorders would be an important milestone and a step towards developing tools for improved diagnostic procedures, identification of high-risk individuals, and personalized psychiatric treatment. Cerebrospinal fluid (CSF) is an especially suitable substrate to use in the quest for biomarkers in psychiatric disorder [1]. Because of its proximity to the central nervous system, CSF reflects the metabolic status of the brain and is analyzed for diagnostic purposes in neurodegenerative disorders such as Alzheimer's and Parkinson's disease [2].

Schizophrenia is presumably a neurodevelopmental disorder characterized by hallucinations, delusions, and cognitive deficits. Bipolar disorder is an affective disorder that presents with episodic mood swings from high periods or mania to low periods of depression, but is also often accompanied by cognitive deficits and psychotic symptoms. Schizophrenia and bipolar disorder have a symptom overlap and in line with this reasoning, epidemiological and genetic studies have suggested a partly shared genetic liability between bipolar disorder and schizophrenia [3], which suggests that these illnesses might share etiological and pathophysiological factors.

In 2002, micrometer-sized structures were identified in CSF from patients with schizophrenia using a scanning electron microscopic (SEM) technique. In 20 of 22 patients with schizophrenia but in only 2 of 38 non-schizophrenic controls micrometer-sized spherical particles were found in CSF [4]. One independent study using frozen CSF samples and different methodologies did not report an association between structures in CSF and schizophrenia [5]. However, in a more recent study of patients with bipolar disorder 45 of 56 patients had thread-like structures, spherical structures, or both vs. none of 20 controls without a psychiatric diagnosis [6]. In the bipolar study the SEM examination was carried out for both a first (the first 0.6 ml) and a second (the next 12 ml) fraction of CSF. Particularly noteworthy is that the structures were more often present in the first than in the second fraction. The microscopic structures were smaller than cells and larger than protein molecules. One hypothesis is that the particles may be related to childhood infections and the subsequent risk of psychotic illness as reported by Dalman et al. in a study of more than one million Swedish subjects [7]. The mainly spherical form points to a high lipid content of the particles, which may reflect an intense apoptosis of lipid rich cell membranes not being cleared rapidly enough by the immune mechanism in the central nervous system. However, the findings may also be accidental or confounded by factors such as medication or comorbidity of psychiatric and somatic disorders. Absence of particles in the controls may also be due to a systematic sampling bias of the CSF collection procedure. Another issue concerns the possibility that the particles are more often found in the first fraction (0.6 mL) of CSF than in the second fraction (12 mL), which bears on the specific techniques used for collecting CSF. If the structures display a rostrocaudal concentration gradient, the first fraction of CSF will reflect the composition of the lumbar dural sac, whereas the second, larger volume of 12 mL reflects the rostral spinal or even ventricular CSF [8].

It is still unknown whether the microscopic patterns identified in the CSF are present in vulnerable but as yet asymptomatic individuals. If this were the case, SEM analysis of CSF could be used to identify individuals with increased risk for the disorders under study [9]. Such a finding would provide biological information about a vulnerability trait, analogue to what has been fruitful in the diagnostics of latent disease carrier in acute intermittent porphyria [10]. One way to elucidate whether latent disease carriers with unexpressed genetic liability also present microscopic structures in CSF is to examine monozygotic and dizygotic twins discordant or concordant for schizophrenia and bipolar disorders. Such an analysis would also provide a possibility to separate genetic and environmental contributions to the development of the manifest diseases [11].

The aim of the present study was to investigate the incidence of microscopic particles in two fractions of fresh cerebrospinal fluid in twin subjects discordant or concordant for schizophrenia or bipolar disorder, unaffected twin pairs, and healthy individuals without major psychiatric disorders. A secondary aim was to examine a large cohort of healthy controls using identical CSF sampling techniques that were used in the study of probands with bipolar disorder [6] to rule out confounding factors due to the collection procedure. We hypothesized that probands with schizophrenia and bipolar disorder, as well as their as yet healthy co-twins at genetic risk for these disorders, would show microscopic particles in CSF more often compared with healthy controls.

## Materials and Methods

### Ethics

The ethical review board of Karolinska Institutet, Stockholm, Sweden (#2007/779) approved the study of the twins, which was performed in compliance with the Helsinki Declaration. The collection of the healthy singleton subjects was part of an ongoing study approved by the Regional Ethics Committee in Stockholm (#2009/1221-32) and conducted in accordance with the latest Helsinki Protocol. All enrolled patients and control subjects consented orally and in writing to participate in the study. There were no adverse effects in the twins or the singletons, which may be explained by fixed routine and that the same neurologist performed the lumbar punctures.

### Selection of twin subjects

Nineteen twin pairs were recruited from a nationwide cohort of Swedish-born, same-sex twins with schizophrenia, bipolar disorder and healthy control-pairs ascertained through the Swedish Twin Registry. In total, 38 twins were included in the study where one or both twins in a pair had an ongoing or past history of bipolar disorder, schizophrenia, or schizoaffective disorder. In four of the included twin-pairs none was affected by bipolar disorder, schizophrenia or schizoaffective disorder. The sibling of one of the 38 recruited twins did not agree to participate in the lumbar puncture. None of the participants was hospitalized at the time of examination. All 37 twins and 65 healthy controls, who received a small remuneration for their participation, consented orally and in writing to take part in the study. The regional ethical review board of Karolinska Institutet, Stockholm, Sweden, approved the study, which was performed in compliance with the Helsinki Declaration.

### Zygosity determination

The zygosity of the twins, mono- or dizygotic, was validated using a robust panel of 47 highly multiplexed single nucleotide polymorphisms (SNPs) that provide reliable and high quality data on a range of different DNA templates [12]. Of the 19 twin pairs recruited for the study, 11 were monozygotic and 8 dizygotic.

### Assessment of twin subjects

All twin individuals were interviewed with The Structured Clinical Interview for DSM-IV Axis I Disorders (SCID-I) [13] and for DSM-IV Axis II Disorders (SCDI-II) [14]. Information about socioeconomic status, smoking habits, and current medication was collected. Psychiatric symptoms were rated using the following scales: Scale for Assessment of Negative Symptoms (SANS) [15], Scale for Assessment of Positive Symptoms (SAPS) [16], Hamilton Depression Rating Scale (HDRS) [17], and Young Mania Rating Scale (YMRS) [18]. The Global Assessment Function (GAF) [19] was used to assess DSM-IV Axis V. Because collection of the CSF in the twin participants was performed two months or more after the psychiatric assessment described above, a psychiatrist performed a complementary psychiatric assessment adjacent to the CSF-collection (LW or VJ) to update information about psychiatric status, current medication and somatic status. Also information about heredity, age of onset, insight, period of active symptoms and life-time somatic diagnosis was collected and psychiatric symptoms were rated using self-report questionnaires of YMRS [18] and Montgomery Asberg Depression Rating Scale (MADRS) [20]. Finally two clinically experienced researchers (CH and VJ) with access to information of the diagnostic assessments, medical records and a full history of lifetime psychiatric diagnostic codes of the Hospital Discharge Registry (1973–2009), but with no access to the CSF-results, decided on a final consensus diagnosis.

### Selection of singleton control subjects

Healthy volunteers were selected randomly for the study by Statistics Sweden (Swedish government agency that produces official statistics of Sweden, www.scb.se) from the National Population Register in the Stockholm area. The participants were matched on sex and age with patients with bipolar disorder enrolled in a prospective study (the St. Göran bipolar project) as previously described [21]. Exclusion criteria applied to the controls were any on-going psychiatric disorder, including bipolar disorder, schizophrenia and schizoaffective disorder, current treatment with any psychotropic drugs, a first-degree relative with schizophrenia, schizoaffective disorder or bipolar disorder. A further exclusion criterion was any condition that precluded magnetic resonance imaging of the brain (e.g., metal implants, shrapnel and certain heart operations). One of the 66 recruited controls was excluded because of a diagnosis of dementia that was revealed during the interview.

### Assessment of singleton control subjects

A research nurse with psychiatric training conducted telephone interviews aimed at screening the participants, whereupon those who fulfilled our screening criteria were scheduled for a visit. At the visit, a psychiatrist interviewed eligible controls in a semi-structured manner (CJE and AJ), using a Swedish modified version of the Affective Disorder Evaluation assessment tool [22]. This interview includes screening for bipolar illness as well as questions about socioeconomic status, use of alcohol and psychoactive substances, family history of psychiatric disorders in first- and second-degree relatives, treatment history and somatic illnesses. The Mini International Neuropsychiatry interview (MINI) was used to screen for psychiatric disorders other than bipolar illness including psychosis and depression [23]. The Global Assessment Function (GAF) [19] was used to assess Axis V. The participants also completed four self-report questionnaires: YMRS [20], MADRS [21], the Alcohol Use Disorders Identification Test (AUDIT) [24] and the Drug Use Disorders Identification Test (DUDIT) [25].

### Blood sampling and CSF/serum albumin ratio and BMI

The blood and CSF investigations for the twins lasted from March 2008 to September 2011 and for the controls from November 2009 to December 2010. Blood samples were collected with the subjects in a fasting state at 08 h under sterile conditions before the lumbar puncture. The integrity of the blood-CSF barrier of the twin and control samples was assessed by the albumin ratio, expressed as CSF albumin (mg/L)/serum albumin (g/L). Blood-CSF barrier dysfunction was defined as an albumin ratio >6.8 in individuals ≤44 years and >10.2 in individuals ≥45 years according to the reference limits presented by Blennow and coworkers [26]. Acute infection or inflammation was defined as high sensitivity C-reactive protein (HS-CRP) >3 mg/L. High level of white blood cell count (WBC) was defined as WBC >8.8×10^9^/L (reference of the hospital laboratory). Height and weight were recorded on the same day as blood and CSF sampling for calculation of the body mass index (BMI) as a heuristic proxy for body fat of the participants.

### Collection of cerebrospinal fluid

The same neurologist performed the 102 lumbar punctures with all subjects in a sitting position. Sixteen twin pairs were examined on the same day and the remaining two pairs within the same month. One of two types of fine disposable needles was used; either Becton Dickinson (BD) 22 GA 3.00 IN, 0.70×75 mm (Quincke needle used in 26 twins and 17 control subjects) or BD Whitaker Needle 25 GA 3.50 IN, 0.50×90 mm (Sprotte needle used in 12 twins and 48 control subjects). The skin in the lumbar region was washed with sterile cotton swabs and chlorhexidine 5 mg/mL (Fresenius Kabi) before puncture. The needles were inserted in vertebral interspace L3 to L4, or L4 to L5, and the very first 12 drops of CSF, approximately 0.6 mL, were collected in a sterile test tube for microscopic examination. The following 12 mL of CSF were allowed to drip spontaneously, or by suction six times using a 2 mL syringe due to slow flow, in a second test tube, which was gently inverted 10 times to secure homogeneous mixing of the components to avoid gradient effects. An aliquot of 0.6 mL of the mixed 12 mL was transferred into a third sterile test tube for immediate filtration and gold coating before microscopic examination. The first and the second fraction of CSF were handled in a similar way for SEM. The last mL of CSF for the control subjects was sent within 30 min after CSF withdrawal to the hospital laboratory for visual inspection and cell counting.

### Filtering of cerebrospinal fluid and filter coating techniques

Of each 0.6 mL fresh CSF fractions, 200 µL were pipetted and dripped onto the surface of a polycarbonate filter (Nucleopore, Inc., Pleasanton, CA, USA) with 0.6 µm pores. The polycarbonate filter was specially prepared by GP Plastic AB (Gislaved, Sweden) and supplied by Sempore AB (Stockholm, Sweden). The filter was fitted to an airtight device designed with flow channels that allows CSF to stream to the center of the filter when vacuum suction is applied from below. This design does not allow particles with sizes larger than 0.6 µm to drip through the filter. The remaining structures in the CSF were thus concentrated in the center of the filter. Once the filter was dried it was coated in a JEOL JFC-1200 Fine Coater (JEOL Tokyo, Japan) during two minutes with ionized gold to a thickness of 40 Å.

### Scanning electron microscopy

**Figure 1:**
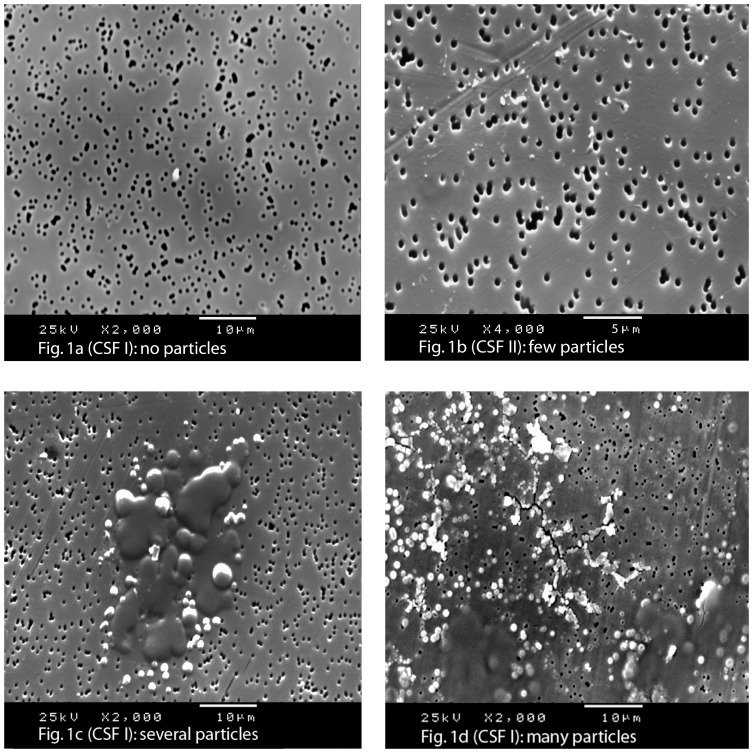
Scanning electron microscopy (SEM) pictures from investigation of fresh cerebrospinal fluid (CSF). Microscopic pictures of representative structures categorized as containing ‘no particles’, ‘few particles’, ‘several particles’ and ’many particles’. A) No particles ×2000, B) few particles ×4000, C) several particles ×2000, and D) many particles ×2000. The CSF is applied on gold plated polycarbonate filters with 0.6 µm pores that may be used for size reference. A) Demonstrates a filter from a healthy singleton control free of spherical particles and with a small peel of skin in the middle. B) The size of the spherical particles from an unaffected co-twin in is about 0.1 µm in diameter similar to the size of microvesicles. C) A filter from a twin with schizoaffective disorder showing spherical particles with the appearance of small lipid bodies, sizes between 0.1 to 8.0 µm, mainly located in the margin of compact aggregates adhering together in a viscous state. D) CSF from a patient with more than 30 years history of schizophrenia, displaying spherical particles ranging from 0.2 to 5 µm in diameter dispersed over a larger part of the filter.

**Figure 2:**
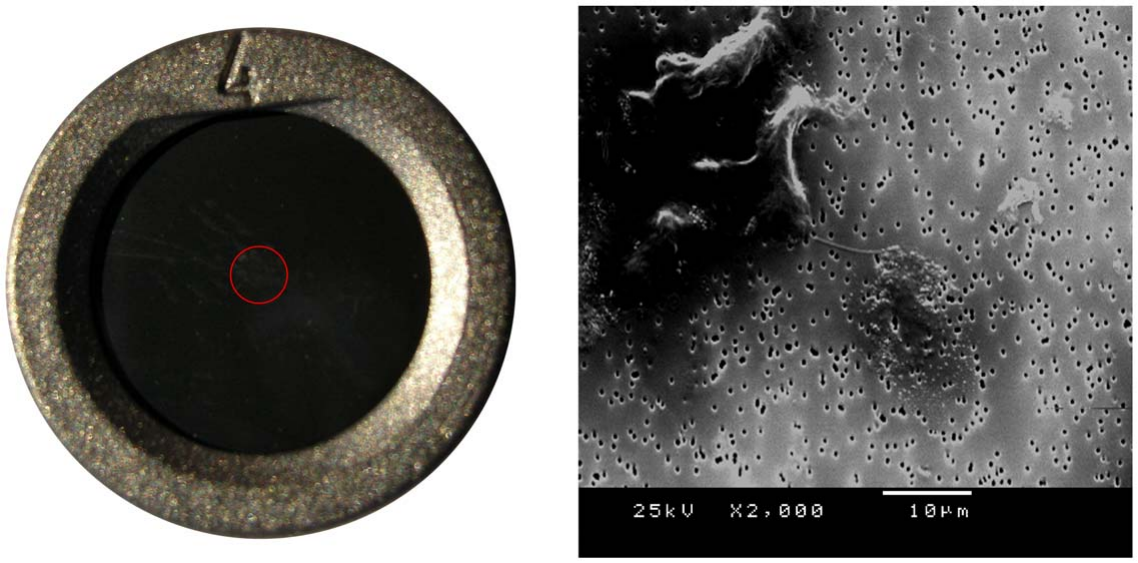
Polycarbonate filter (left) and scanning electron microscopy (SEM) picture from a healthy control (right). To the left a photo of a polycarbonate filter with 10 mm diameter and 0.6 µm pores fitted to an airtight device. The red ring in the centre indicates the area where the morphological structures are concentrated during the vacuum suction after 200 µL of cerebrospinal fluid (CSF) has been applied on top of the filter. To the right a SEM picture (×2000) from a healthy control. She was the only one of the 65 healthy controls displaying particles her CSF. In the upper left corner is a cell of phagocyte type with a chord extending towards a larger cluster of spherical particles with a diameter of 0.1–0.2 µm located in the center of the filter.

The 204 filters in the present study were analyzed in a SEM microscope (Philips High Resolution SEM 515, Philips Electronic Instruments, Eindhoven, The Netherlands). The total area of each filter with a diameter of one cm was examined in the microscope and rated by an experienced researcher (RN). The peripheral area outside the center was mostly free of structures. To standardize the procedure, SEM images of the central areas of the filters were enlarged 50, 500 and 2000 times and saved for further reference. A second researcher (LW) rated the same images with similar results. The microscopic quantity of the morphological structures on each filter was rated in the following four categories: 0 = no, 1 = few, 2 = several and 3 = many structures (Fig. 1) as described previously by Båve et al with good interrater consistency [6]. Particles larger than 0.6 µm and some with smaller diameters (down to 0.1 µm) were revealed (Fig. 1 and Fig. 2).

### Blood sampling and CSF/serum albumin ratio

Blood samples were collected under sterile conditions before the lumbar puncture. The integrity of the blood-CSF barrier of the twin and control samples was assessed by the albumin ratio, expressed as CSF albumin (mg/L)/serum albumin (g/L). Blood-CSF barrier dysfunction was defined as an albumin ratio >6.8 in individuals ≤44 years and >10.2 in individuals ≥45 years according to the reference limits presented by Blennow and coworkers [26]. Acute infection or inflammation was defined as high sensitivity C-reactive protein (HS-CRP) >3 mg/L. High level of white blood cell count (WBC) was defined as WBC >8.8×10^9^/L (reference of the hospital laboratory).

### Diagnostic categories

The results of the clinical assessment of the 37 twins are presented in Table 1. All patients with bipolar disorder type I had a history of one or more psychotic episodes confirmed by ICD-diagnosis information in register data. The cases diagnosed with schizophrenia, schizoaffective disorder or bipolar disorder are referred to as “proband”, whereas the healthy co-twins from the discordant twin pairs are referred to as “co-twin”. The twins and controls were categorized into three subgroups for statistical analysis (Table 2).

**Table 1:** Diagnostic classification in 37 twins participating in the CSF study.

Diagnostic status	Twin subjects		Twins from concordant pair*	Twins from discordant pair	Twins from control pair
	MZ twins	DZ twins			
Schizophrenia	4	3	5	2	-
Schizoaffective disorder	1	1	0	2	-
Bipolar disorder type 1	4	1	1	4	-
Bipolar disorder type 2	1	2	0	3	-
Life-time depression	6	3	0	5	4
Not affected	5	6	0	7	4
Total	21	16	6	23	8

* Classified as concordant when both individuals in the pair were affected by schizophrenia, schizoaffective disorder or bipolar disorder.

**Table 2:** Subgroups for analysis of CSF data and the distribution of the diagnostic categories.

Group status	Schizophrenia	Schizoaffective disorder	Bipolar disorder	Total
Probands	7	2	8	**17**
Co-twins	3*	2*	7*	**12**
Healthy controls and unaffected twins**	-	-	-	**73**

* Indicates the disease status of the proband, 11 complete discordant twin pairs in total.

** In total 65 singletons and 4 complete twin pairs.

### Statistical analysis

Descriptive statistics are expressed as means (standard deviations) or medians (minimum and maximum values) for continuous variables and frequencies (percentages) for categorical variables. Group differences in our data were examined using the exact conditional logistic regression model [Proc Logistic, EXACT-statement, Statistical Analysis Software (SAS 9.3)] [27] and non-independency within twin-pairs was controlled for in a separate analysis by removing either twin 1 or twin 2 from the disease concordant pairs in the proband-group and from the healthy twin-pairs in the control-group. The results were expressed as odds ratios (ORs) and Wald's confidence limits (CLs) and a two-sided p-value<0.05 was considered to be statistically significant. Group status entered the model as a dependent variable and any quantity of structures in the first or second fraction of CSF as an independent variable. Age, sex, BMI, lifetime alcohol abuse or dependence, lifetime anxiety syndrome, any medical disorder and smoking status entered the model as covariates. Albumin ratio was categorized as normal or as an indicator of blood-CSF barrier dysfunction if the ratio exceeded the reference limit and entered the model as a covariate. Likewise HS-CRP and WBC were categorized as normal or above the reference limit and entered the model as covariates. The effect of psychotropic medication was assessed using logistic regression models with the CSF finding as the dependent variable and medication (along with age and sex) as the independent variable. Subgroups of psychotropic medications were tested as follows: neuroleptics, lithium, anticonvulsants and antidepressants. Fisher's exact test was used to examine any association between a positive CSF finding and zygosity.

## Results

### Structures in CSF identified by SEM

Structures of the CSF were examined on two filters from each participant, one from the first and one from the second sample of fresh CSF. Spherical particles were noted with the appearance of small lipid bodies averaging between 0.1 to 8.0 µm. Filters with a negative finding did not present any structures (Fig. 1 A). Particles with sizes down to a diameter of 0.1 µm were found both separately on the filter (Fig. 1 B) and in the margin of larger aggregates adhering together in a viscous state (Fig. 1 C) or as separate aggregates between 0.2 to 5.0 µm (Fig. 1 D). Microscopic pictures of representative structures categorized as containing ‘no particles’, ‘few particles’, ‘several particles’ and ‘many particles’ in the first or second fraction of CSF are depicted in Fig. 1 a–d. In total 12 of 17 probands, 5 of 12 co-twins and 3 of 73 healthy controls presented particles in their CSF.

### Association between CSF-structures and group-status

Demographic data are presented in Table 3. The distribution of positive CSF findings in the first or the second fraction in probands, co-twins, and controls are seen in Table 4 along with the logistic regression analysis with the control group as a reference, and age and sex as covariates. The results showed that a positive microscopic finding in CSF significantly increased the probability of being a proband or a co-twin compared to the rate observed in the control group. When analyzing the results of the first and the second CSF-fraction separately, the results were still significant when analyzing only the first fraction. Seven individuals displayed particles in the second fraction of CSF; six of them belonged to the proband group, one to the co-twin group, and all 7 individuals displayed a positive finding also in the first CSF fraction. The results of quantification of morphological structures in the categories ‘no’, ‘few’, ‘several’ and ‘many’ in the first CSF fraction (Table 5) and the second CSF fraction (Table 6) are presented in the diagnostic categories. For detailed information on all participants see Table S1.

**Table 3:** Sample demographics and laboratory data of 102 subjects included in the study.

Demographic data	Probands, N = 17	Co-twins, N = 12	Control-twins/singletons, N = 73
Age (years, mean ± SD)	49.5±12.1	51.1±10.6	39.7±14.3
Sex (female %)	58.8	66.7	57.5
Cohabitation status (%) a	23.5	41.7	64.4
Higher education (%) b	35.3	50.0	45.2
Occupation status (%) c	29.4	75.0	89.0
Current medication (%) d	82.4	41.7	16.4
Current psychotropic medication (%) d	76.5	25.0	2.7*
Previous/current somatic illness (%)	41.2	50.0	20.6
Previous alcohol abuse/dependence (%)	41.1	8.3	1.4
Previous history of anxiety syndrome (%)	41.1	8.3	11.0
Smoking (%)	47.1	8.3	15.1
Snuff user (%)	29.4	8.3	11.0
Body mass index (mean ± SD)	28.0±7.8	27.5±6.0	24.2±3.6
Global Assessment of Functioning [median (max-min)]	55 (35–80)	75 (50–100)	80 (70–92)

Laboratory data			
Albumin ratio (mean ± SD)	5.8±1.5	4.6±1.9	5.1±2.3
White blood cell count (mean ± SD)	6.5±2.0	5.9±1.3	5.7±1.7
C-reactive protein (mean ± SD)	2.3±3.0	4.1±5.6	2.6±4.8 (n = 66)

a ) Defined as living with partner/family.

b ) Defined as studies on University level.

c ) Defined as employment in the open labor.

d ) Defined as prescribed medication taken daily.

* A twin pair unaffected by schizophrenia or bipolar disorder were on antidepressants at the time of lumbar puncture.

**Table 4:** Distribution of scanning electron microscopic findings in cerebrospinal fluid (CSF) and results from the logistic regression analysis.

Group status and N	Positive CSF findings (%)	Odds ratio*	Confidence limits	p-value
Probands (N = 17)	12 (70.6)	48.5	8.2–550.8	<0.0001
Co-twins (N = 12)	5 (41.7)	16.2	2.0–217.8	0.006
Healthy controls and unaffected twins (N = 73)	3 (4.1)	Reference	-	-
Total (N = 102)	20 (19.6)			

* Logistic regression analysis with age and sex as covariates.

**Table 5:** First fraction of cerebrospinal fluid (0.2 mL of 0.6 mL) examined with scanning electron microscope in twins and singleton controls.

Diagnostic groups	Amount of particles in the first cerebrospinal fluid fraction			
	No	Few	Several	Many
Schizophrenia	0	4	1	2
Schizoaffective disorder	1	0	1	0
Bipolar disorder type I	1	3	1	0
Bipolar disorder type II	3	0	0	0
Depression	11	3	1	0
Unaffected	66	4	0	0
Total	82	14	4	2

**Table 6:** Second fraction of cerebrospinal fluid (0.2 mL of 12 mL) examined with scanning electron microscope in twins and singleton controls.

Diagnostic groups	Amount of particles in the second cerebrospinal fluid fraction			
	No	Few	Several	Many
Schizophrenia	3	2	0	2
Schizoaffective disorder	1	1	0	0
Bipolar disorder type I	4	1	0	0
Bipolar disorder type II	3	0	0	0
Depression	14	1	0	0
Unaffected	70	0	0	0
Total	95	5	0	2

A series of analyses were conducted in order to determine whether a history of alcohol abuse or anxiety syndrome, somatic disorder, smoking, blood-CSF-barrier dysfunction (high albumin ratio), BMI, type of needle used in the lumbar puncture procedure, acute infection or inflammation and use of psychotropic medications could account for the above associations. A previous or ongoing history of alcohol abuse or dependency strongly predicted the risk of being a proband (OR = 89.8, CL 3.1>999, p<0.009). However, when we tested the effect of a positive CSF finding among the probands and controls with no history of alcohol abuse or dependency (probands n = 10 and controls n = 72), microscopic particles in the CSF continued to be associated with proband status (OR = 131, CL 9.0>999, p<0.0001). Anxiety syndrome, somatic disorder, smoking and type of needle at lumbar puncture (Quincke vs. Sprotte needle) did not predict the risk of being a proband or a co-twin. Including high vs. low levels of albumin ratio in the model did not change the significance of the associations between CSF particles and proband or co-twin status. When individuals (n = 7) with a high albumin ratio - indicating a defect blood-CSF-barrier - were removed from the analysis, the association between proband/co-twin status and a positive CSF finding remained significant.

### Indicators of infection and inflammation

Since acute or chronic infection may be a cause of the structures in CSF, we investigated measures of acute and chronic infection or inflammation. HS-CRP and WBC were used as indicators of acute infection. Excluding subjects from the analysis with HS-CRP or WBC above the reference limit did not change the significance of the associations of CSF particles with the proband or co-twin status. The effect of a previous infection, chronic rheumatic disorder, or gastro-inflammatory disorder were included separately in the model but did not affect the association between proband or co-twin status and microscopic particles.

### Psychotropic medication and statins

The effect of psychotropic medication on the microscopic CSF findings was tested in the proband group, since the non-proband subjects had not received psychotropic medications with the exception of three co-twins and two control-twins treated with antidepressants at the time of lumbar puncture. Neither “any psychotropic medication” (OR = 5.43, CL 0.51-infinity, p<0.24), nor any of the separately tested psychotropic medications [neuroleptics (OR = 4.0, CL 0.4–120 p<0.32), lithium (OR = 0.39, CL 0–7.3 p<0.56), anticonvulsants (OR = 0.27, CL 0–3.73 p<0.40)] had a significant effect on the presence of CSF structures. The effect of antidepressants was tested on the individuals with a diagnosis of schizophrenia, schizoaffective disorder, bipolar disorder, and depression (OR = 1.1, CL 0.14–8.86, p<1).

Six individuals, five twins and one healthy singleton were prescribed statins (cholesterol-lowering drugs) of whom three displayed microscopic CSF particles.

### Association between CSF findings and zygosity

To explore a possible genetic contribution to the CSF results, we tested the effect of zygosity in the co-twin group. There was a clear tendency for a positive CSF finding to be more frequent in the MZ co-twins (57%) than in the DZ co-twins (20%), but this difference did not reach significance in this limited sample of twins (Fisher's Exact Test: F = 3, p = 0.22).

### Association between CSF-structures and different diagnostic categories

Detailed analyses were performed in the sample to investigate the association between particles in the CSF and the separate diagnostic groups divided into schizophrenia or schizoaffective disorder and bipolar disorder type I or type II. Microscopic particles were strongly associated to schizophrenia and schizoaffective disorder (OR = 67.9, CL 13.6–999, p<0.0001, probands n = 9 and controls n = 73) and their co-twins (OR = 41.5, CL 6.2–999, p<0.0007, co-twins n = 7 and controls n = 73). Moreover particles were associated to bipolar disorder type I and II (OR = 26.5, CL 2.3–999, p<0.004, probands n = 8 and controls n = 73), but not to their respective co-twins (OR = 3.1, CL 0.04–69.4, p<0.8, co-twins n = 7 and controls n = 73).

In the group of patients with bipolar disorder type I, all subjects had experienced at least one psychotic episode. We therefore analyzed the association between particles and patients with psychotic features including probands with schizophrenia, schizoaffective disorder and bipolar disorder type 1, removing the patients and co-twins with bipolar disorder type II. The association continued to be significant in probands (OR = 90.9, CL 12.2–999, p<0.0001, probands n = 14 and controls n = 73) and co-twins (OR = 18.7, CL 2.6>199.6 p<0.0023, co-twins n = 9 and controls n = 73).

The association between CSF-particles and group-status when excluding the subjects with a history of lifetime depression in the control-group increased in the proband group (OR = 163.1, CL 13.4–999, p<0.0001, probands n = 17 and controls n = 61) as well as in co-twins (OR = 51.5, CL 3.6–999, p<0.0009 co-twins n = 12 and controls n = 61).

### Association between CSF-structures and group-status considering non-independency within twin-pairs

We performed a separate analysis to compensate for non-independency within twin-pairs by removing either twin 1 or twin 2 from the disease concordant pairs in the proband group and from the healthy twin-pairs in the control-group. When we tested the effect in probands (n = 14) and co-twins (n = 12) compared to controls (n = 69) particles in CSF continued to be associated with proband status (OR 51.9 CL 6.7–877.3, p<0.0001) and co-twin status (OR 23.3 CL 2.4–399.7, p = 0.003). We got the same results when removing twin 1 as when removing twin 2.

### Description of control subjects with a positive finding in CSF

**Figure 3:**
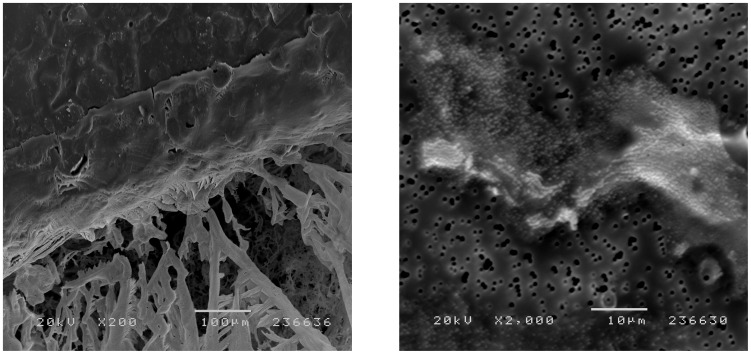
Scanning electron microscopy (SEM) picture of cerebrospinal fluid (CSF) from monozygotic twins unaffected by psychotic disorder. SEM pictures of a polycarbonate filter with the first fraction of CSF from a monozygotic twin pair unaffected by psychotic illness, but with a medical history of episodic depressions and atrial fibrillation. Both twins displayed the highest CSF albumin concentration in the study. The twin in figure A) also had a history of Parkinson's disease treated with L-dopa. Figure B) contains subcellular structures with spherical shapes.

**Figure 4:**
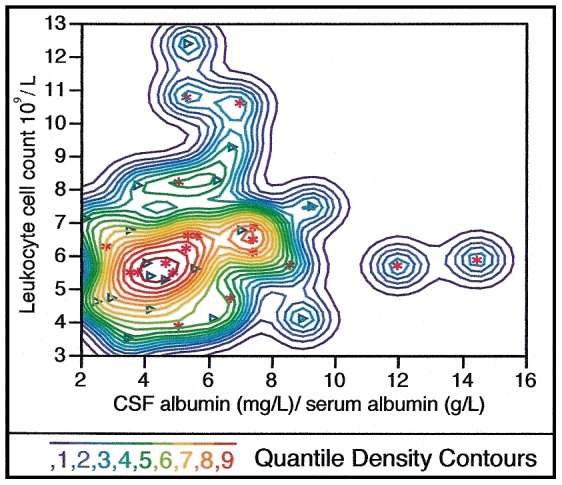
Albumin ratio CSF/serum versus blood leukocytes in 37 twin subjects. A nonparametric bivariate density graphic plot of the albumin ratio expressed as cerebrospinal fluid (CSF) albumin (mg/L)/serum albumin (g/L) on the ordinate vs. the leukocyte number ×10^9/^L on the abscissa in 37 twins. To the right are two outliers with a high albumin ratio, indicating that they have a defect in their blood-CSF barrier function (for individual albumin ratio see Table S1 in Supporting Information).

Three individuals assigned to the healthy controls and unaffected twins had structures in their CSF (Table 4). One female singleton control under the age of 45 years displayed particles in her CSF and was the only one with a positive CSF finding from the 65 singleton controls. She was apparently healthy with no medication, had no family history of psychiatric disorders and her CSF/serum albumin ratio was within the normal reference range. The SEM picture of her first CSF fraction reveals a chord extending towards a larger cluster of spherical particles with a diameter of 0.1–0.2 µm located in the center of the filter (Fig. 2). Two male participants from a monozygotic twin pair with a history of depression and anti-depressive treatment displayed particles in the first fraction of CSF (Fig. 3 A, B). Both participants had a previous history of depression, ongoing antidepressive treatment, increased CSF/serum albumin ratio (14.5 and 12.0) and a medical history of atrial fibrillation (Fig. 4). The first twin was affected with Parkinson's disease, had a normal WBC (5.9×10^9^/L), an increased level of HS-CRP (16.9 mg/L) and protein-like aggregates were observed in his CSF sample (Fig. 3 A). The second twin had a normal level of WBC (5.7×10^9^/L), a slightly increased level of HS-CRP (3.5 mg/L) and displayed spherical particles in his CSF (Fig. 3 B).

## Discussion

We analyzed CSF in 37 twins and 65 controls using the SEM technique to identify potential genetic and environmental effects influencing accumulation of particles in CSF. The main findings from this report are as follows.

First, the structures in CSF were strongly associated with schizophrenia and bipolar disorder. These results confirm previous findings in schizophrenia [4] and bipolar disorder [6].

Second, the structures were rare in the healthy controls, implying that structures detected with this method are exceptional in the normal population. The CSF of the twins and controls were sampled following identical procedures, which were also used in a previous study investigating bipolar disorder [6], which rules out the possibility that the findings were due to different sampling techniques.

Third, we found an association between structures in the CSF and co-twin status, which indicates a genetic or environmental effect. Control for potential confounding factors in the logistic regression models suggests that the CSF structures do not seem to be explained by medication, alcohol abuse, anxiety disorder, somatic disorder, BMI or type of needle used during lumbar puncture.

Finally, we studied the distribution of CSF structures in the monozygotic and dizygotic co-twins and observed a clear trend towards a higher level of positive CSF findings in the monozygotic co-twins. These results did not reach the statistical threshold, probably because of a lack of power due to small sample size. Taken together the findings are consistent with a genetic influence on the CSF findings.

CSF/serum albumin ratio has been suggested as a marker of blood-CSF barrier dysfunction [26], [28]. Earlier studies have reported on increased ratio in some patients with schizophrenia [29], [30]. However, none of the participants with schizophrenia or bipolar disorder in our sample had an increased albumin ratio. Thus, when included in the model blood-CSF barrier dysfunction, did not have any effect on the main results. We also included markers for acute inflammatory activity, current medication and information about somatic disorders, (e.g., inflammatory-related disorders and neurological disorders) in the analysis. The results support the assumption that the presence of CSF structures is not sensitive to ongoing infection or inflammation. Investigating the presence of previous infections, we decided not to include reports of unspecified virus infections in that they are very common and unspecific. The effect of psychotropic medication (antipsychotics, lithium, anticonvulsants and antidepressants), examined in the proband group only did not show any effect on the presence of CSF particles when tested separately or combined. This finding contradicts the view that CSF structures are related to psychotropic medication.

When analyzing the probands with schizophrenia and schizoaffective disorder and their co-twins separately from the probands with bipolar disorder and their co-twins we found a stronger association between schizophrenia and schizoaffective disorder and particles in CSF compared to bipolar disorder. There was no significant association between the co-twins discordant for bipolar disorder and particles. However when we analyzed without the patients with bipolar disorder type II and their co-twins the association between group-status and particles increased compared to our main results indicating that the particles in CSF may be associated with psychotic symptoms. This finding is in line with the tendency reported in the study by Båve et al. in which patients with bipolar disorder type I were more likely to display CSF structures than patients with bipolar II disorder [6].

In 13 of the 20 individuals with a positive CSF-finding, structures were identified in the first 0.6 ml of CSF but not the second fraction. A consensus guideline for neurological research from 2009 prescribes that the very first mL of CSF should be discarded [31]. Interestingly following this recommendation would only have identified individuals with CSF structures in the second fraction. One hypothesis is that a low level of neuroinflammation or neurodegeneration in schizophrenia and bipolar disorder gives rise to a smaller amount of apoptotic products in the CSF. The individuals who also display structures in the second CSF fraction may have numerous particles in their CSF, indicating that they have a less effective cleansing function of CSF or a higher level of neurodegeneration in the brain [8], [32]. The possibility that the structures are unspecific artifacts does not accord with the negative findings in the controls.

Only 3 controls presented structures in their CSF. One female singleton control presented spherical particles in her CSF of a similar type found in patients with schizophrenia and bipolar disorder (Fig. 2). Particles of similar size and form were found in the second CSF fraction of an unaffected, un-medicated male co-twin of a brother with schizoaffective disorder (Fig. 1 B). Further, a monozygotic twin pair that served as controls presented particles in their CSF (Fig. 3). The structures observed in the first twin are similar to protein-like aggregates, possibly related to the increased albumin ratio in CSF (Fig. 3 A). The structures in the second twin were similar to the spherical particles observed in the proband sample (Fig. 3 B). The findings in both twins may be related to a blood-CSF barrier dysfunction. They may also be latent disease carriers for bipolar disorder or schizophrenia.

Furthermore, spherical CSF-particles may also occur in other disorders involving the central nervous system as recently reported in amyotrophic lateral sclerosis by Zachau et al. [33]. In this study the CSF of an ALS patient displayed 100 times more phosphatidylserine-positive microparticles and 400 times more cell-derived microparticles of leukocyte origin compared to healthy controls although the leukocyte count in CSF was normal. Similar studies of microparticles in CSF in schizophrenia and bipolar disorders are called for.

### Strengths and limitations

Strengths of the present study include the careful lifetime assessment of the psychiatric diagnosis, controlling for potential confounding factors in the analysis and an identical collection procedure of the CSF in all participants included in the study. Moreover, the control group is large and population based. However, several limitations of the study also need to be addressed. The possible confounding effects of perinatal and childhood infections were not clarified because of lack of information. The small sample size of disease discordant twin-pairs prevented further analysis of the genetic contribution of the CSF findings. In addition, psychotic symptoms of the probands were not investigated using a structured instrument such as SANS and SAPS [15], [16] adjacent to the collection of CSF.

### Further studies

Further research in larger twin samples of cases diagnosed with schizophrenia and bipolar disorder as well as high-risk individuals (e.g., first-degree relatives of patients with psychosis) may help to answer the question of whether structures of the CSF precede a manifest disease state and may serve as a trait marker. Further work is needed to explore the origin and the composition of the anatomical structures in both the first and second CSF fractions.

### Conclusions

The main conclusion from this study is the strong statistical support for structures in CSF identified with the SEM technique in patients with bipolar disorder or schizophrenia. The results indicate that the CSF structures likely originate from trait-dependent factors.

## Supporting Information

Table S1 **Detailed information on the characteristics of 102 individuals included in the study.**
(DOC)
