# Supplementary material for: Microscopic Particles in Two Fractions of Fresh Cerebrospinal Fluid in Twins with Schizophrenia or Bipolar Disorder and in Healthy Controls
Source: PLoS One. 2012 Sep 25;7(9):e45994. doi: 10.1371/journal.pone.0045994 (PMC3457946; doi:10.1371/journal.pone.0045994)
Supplement: Table S1 — Detailed information on the characteristics of 102 individuals included in the study. (DOC) [file pone.0045994.s001.doc]

**Table S1. Detailed information on the characteristics of 102 individuals included in the study**

| **No** | **Pair no** | **Sex^1^** | **Age^2^** | **Zygosity^3^** | **Diagnosis** | **Concordance** | **Group status** | **CSF-Result^4^** | **CSF 1st^5^ and 2nd^6^ fraction** | | **Albumin Ratio^7^** | **Neuroleptic treatment** | **Anti-convulsive treatment** | **Lithium treatment** | **Anti-depressive treatment** | **Somatic medication** | **Life-time Alcohol Problem^8^** | **Life-time Anxiety Disorder^9^** | **Type of needle LP^10^** | **BMI^11^** |
| --- | --- | --- | --- | --- | --- | --- | --- | --- | --- | --- | --- | --- | --- | --- | --- | --- | --- | --- | --- | --- |
| 1 | 1 | F | >=45 | MZ | Schizophrenia | Concordant | Proband | 1 | 3 | 3 | 5.3 | Yes | No | No | No | No | No | No | Quincke | 26 |
| 2 | 1 | F | >=45 | MZ | Schizophrenia | Concordant | Proband | 1 | 3 | 3 | 4.7 | Yes | No | No | No | No | Yes | Yes | Quincke | 22 |
| 3 | 2 | F | >=45 | MZ | Bipolar I | Discordant | Proband | 1 | 1 | 0 | 5.1 | Yes | No | No | Yes | Yes | Yes | No | Quincke | 42 |
| 4 | 3 | M | >=45 | DZ | Schizophrenia | Concordant | Proband | 1 | 1 | 0 | 7.5 | No | No | No | No | No | Yes | No | Quincke | 20 |
| 5 | 3 | M | >=45 | DZ | Schizophrenia | Concordant | Proband | 1 | 1 | 0 | 5.1 | Yes | No | No | No | No | No | Yes | Quincke | 26 |
| 6 | 5 | M | >=45 | DZ | Schizoaffective | Discordant | Proband | 0 | 0 | 0 | 7.1 | Yes | Yes | Yes | Yes | Yes | Yes | No | Quincke | 38 |
| 7 | 7 | F | >=45 | MZ | Schizophrenia | Discordant | Proband | 1 | 1 | 1 | 8.6 | Yes | No | No | Yes | Yes | Yes | Yes | Quincke | 28 |
| 8 | 11 | F | <45 | DZ | Bipolar I | Discordant | Proband | 0 | 0 | 0 | 3.8 | Yes | Yes | No | Yes | Yes | Yes | Yes | Quincke | 46 |
| 9 | 12 | F | >=45 | MZ | Bipolar I | Discordant | Proband | 0 | 0 | 0 | 5.6 | No | No | Yes | No | No | No | No | Quincke | 28 |
| 10 | 13 | F | >=45 | DZ | Bipolar I | Discordant | Proband | 1 | 1 | 0 | 7 | No | No | Yes | Yes | No | No | Yes | Quincke | 22 |
| 11 | 14 | F | >=45 | MZ | Bipolar I | Discordant | Proband | 0 | 0 | 0 | 9 | No | No | No | No | Yes | No | No | Quincke | 24 |
| 12 | 15 | M | >=45 | MZ | Bipolar I | Discordant | Proband | 1 | 1 | 0 | 5.3 | No | No | Yes | No | Yes | No | No | Quincke | 34 |
| 13 | 16 | F | >=45 | DZ | Bipolar I | Discordant | Proband | 0 | 0 | 0 | 4.2 | No | No | No | No | No | Yes | No | Sprotte | 30 |
| 14 | 17 | F | >=45 | DZ | Schizophrenia | Discordant | Proband | 1 | 2 | 0 | 5.4 | Yes | No | No | No | Yes | No | No | Sprotte | 20 |
| 15 | 18 | M | <45 | MZ | Schizoaffective | Discordant | Proband | 1 | 2 | 1 | 3.8 | Yes | Yes | No | No | No | No | No | Sprotte | 26 |
| 16 | 19 | M | <45 | MZ | Schizophrenia | Concordant | Proband | 1 | 1 | 1 | 4.9 | No | No | No | No | No | No | Yes | Sprotte | 24 |
| 17 | 19 | M | <45 | MZ | Bipolar I | Concordant | Proband | 1 | 2 | 1 | 5.7 | No | No | Yes | No | No | No | Yes | Sprotte | 20 |
| 18 | 2 | F | >=45 | MZ | None | Discordant | Co-twin | 1 | 1 | 0 | 7.4 | No | No | No | No | No | No | No | Quincke | 34 |
| 19 | 5 | M | >=45 | DZ | None | Discordant | Co-twin | 1 | 1 | 0 | 2.8 | No | No | No | No | Yes | Yes | No | Quincke | 24 |
| 20 | 7 | F | >=45 | MZ | None | Discordant | Co-twin | 1 | 1 | 0 | 7.4 | No | No | No | No | Yes | No | No | Quincke | 30 |
| 21 | 8 | M | >=45 | MZ | Depression | Discordant | Co-twin | 1 | 1 | 0 | 6.7 | No | No | No | No | No | No | No | Quincke | 18 |
| 22 | 11 | F | <45 | DZ | Depression | Discordant | Co-twin | 0 | 0 | 0 | 2.2 | No | No | No | Yes | No | No | No | Quincke | 38 |
| 23 | 12 | F | >=45 | MZ | Depression | Discordant | Co-twin | 0 | 0 | 0 | 3.5 | No | No | No | Yes | No | No | No | Quincke | 36 |
| 24 | 13 | F | >=45 | DZ | Depression | Discordant | Co-twin | 0 | 0 | 0 | 6.3 | No | No | No | Yes | Yes | No | No | Quincke | 28 |
| 25 | 14 | F | >=45 | MZ | None | Discordant | Co-twin | 0 | 0 | 0 | 4.7 | No | No | No | No | No | No | No | Quincke | 24 |
| 26 | 15 | M | >=45 | MZ | None | Discordant | Co-twin | 0 | 0 | 0 | 4.1 | No | No | No | No | No | No | Yes | Sprotte | 26 |
| 27 | 16 | F | >=45 | DZ | None | Discordant | Co-twin | 0 | 0 | 0 | 3.6 | No | No | No | No | No | No | No | Sprotte | 26 |
| 28 | 17 | F | >=45 | DZ | None | Discordant | Co-twin | 0 | 0 | 0 | 2.5 | No | No | No | No | No | No | No | Sprotte | 22 |
| 29 | 18 | M | <45 | MZ | Depression | Discordant | Co-twin | 1 | 2 | 1 | 3.6 | No | No | No | No | No | No | No | Sprotte | 24 |
| 30 | 4 | M | >=45 | MZ | Depression | Control twin | Control | 0 | 0 | 0 | 6.8 | No | No | No | No | No | No | No | Quincke | 26 |
| 31 | 4 | M | >=45 | MZ | None | Control twin | Control | 0 | 0 | 0 | 5.4 | No | No | No | No | No | No | No | Quincke | 24 |
| 32 | 6 | M | >=45 | DZ | Depression | Control twin | Control | 0 | 0 | 0 | 9.3 | No | No | No | No | No | Yes | No | Quincke | 28 |
| 33 | 6 | M | >=45 | DZ | None | Control twin | Control | 0 | 0 | 0 | 6.2 | No | No | No | No | Yes | No | No | Quincke | 24 |
| 34 | 9 | M | >=45 | MZ | Depression | Control twin | Control | 1 | 1 | 0 | 14.5* | No | No | No | Yes | Yes | No | Yes | Quincke | 22 |
| 35 | 9 | M | >=45 | MZ | Depression | Control twin | Control | 1 | 1 | 0 | 12* | No | No | No | Yes | Yes | No | No | Quincke | 22 |
| 36 | 10 | M | >=45 | DZ | None | Control twin | Control | 0 | 0 | 0 | 4.2 | No | No | No | No | No | No | No | Quincke | 24 |
| 37 | 10 | M | >=45 | DZ | None | Control twin | Control | 0 | 0 | 0 | 3 | No | No | No | No | Yes | No | No | Quincke | 26 |
| 38 | . | M | <45 | . | None | Control | Control | 0 | 0 | 0 | 3.5 | No | No | No | No | No | No | No | Sprotte | 24 |
| 39 | . | F | <45 | . | None | Control | Control | 0 | 0 | 0 | 4.1 | No | No | No | No | No | No | No | Sprotte | 26 |
| 40 | . | M | <45 | . | None | Control | Control | 0 | 0 | 0 | 7.5* | No | No | No | No | No | No | No | Sprotte | 30 |
| 41 | . | M | <45 | . | Depression | Control | Control | 0 | 0 | 0 | 5.7 | No | No | No | No | No | No | No | Sprotte | 24 |
| 42 | . | F | <45 | . | Depression | Control | Control | 0 | 0 | 0 | 2.6 | No | No | No | No | No | No | No | Sprotte | 28 |
| 43 | . | F | <45 | . | None | Control | Control | 0 | 0 | 0 | 3 | No | No | No | No | No | No | No | Sprotte | 22 |
| 44 | . | F | <45 | . | None | Control | Control | 0 | 0 | 0 | 4.1 | No | No | No | No | No | No | No | Quincke | 30 |
| 45 | . | F | <45 | . | None | Control | Control | 0 | 0 | 0 | 9.7* | No | No | No | No | No | No | No | Sprotte | 24 |
| 46 | . | F | <45 | . | None | Control | Control | 0 | 0 | 0 | 3.3 | No | No | No | No | No | No | No | Sprotte | 22 |
| 47 | . | M | <45 | . | None | Control | Control | 0 | 0 | 0 | 3.9 | No | No | No | No | No | No | No | Sprotte | 18 |
| 48 | . | F | <45 | . | None | Control | Control | 0 | 0 | 0 | 3.5 | No | No | No | No | Yes | No | No | Sprotte | 20 |
| 49 | . | F | <45 | . | None | Control | Control | 0 | 0 | 0 | 4.6 | No | No | No | No | No | No | No | Sprotte | 22 |
| 50 | . | F | <45 | . | None | Control | Control | 0 | 0 | 0 | 3.8 | No | No | No | No | No | No | No | Quincke | 20 |
| 51 | . | M | <45 | . | Depression | Control | Control | 0 | 0 | 0 | 5.7 | No | No | No | No | No | No | Yes | Sprotte | 30 |
| 52 | . | F | <45 | . | None | Control | Control | 0 | 0 | 0 | 4.4 | No | No | No | No | No | No | No | Sprotte | 22 |
| 53 | . | F | <45 | . | None | Control | Control | 0 | 0 | 0 | 3.3 | No | No | No | No | No | No | No | Sprotte | 24 |
| 54 | . | M | <45 | . | None | Control | Control | 0 | 0 | 0 | 5 | No | No | No | No | No | No | No | Sprotte | 26 |
| 55 | . | F | <45 | . | None | Control | Control | 0 | 0 | 0 | 6 | No | No | No | No | No | No | No | Sprotte | 20 |
| 56 | . | M | >=45 | . | None | Control | Control | 0 | 0 | 0 | 5.4 | No | No | No | No | No | No | No | Quincke | 24 |
| 57 | . | M | <45 | . | None | Control | Control | 0 | 0 | 0 | 6.5 | No | No | No | No | No | No | No | Quincke | 28 |
| 58 | . | M | <45 | . | None | Control | Control | 0 | 0 | 0 | 3.4 | No | No | No | No | No | No | No | Sprotte | 24 |
| 59 | . | F | <45 | . | None | Control | Control | 0 | 0 | 0 | 3.3 | No | No | No | No | Yes | No | No | Sprotte | 28 |
| 60 | . | F | <45 | . | None | Control | Control | 0 | 0 | 0 | 5.3 | No | No | No | No | No | No | No | Quincke | 40 |
| 61 | . | F | <45 | . | None | Control | Control | 0 | 0 | 0 | 6.8 | No | No | No | No | No | No | No | Sprotte | 22 |
| 62 | . | F | <45 | . | None | Control | Control | 0 | 0 | 0 | 3.6 | No | No | No | No | No | No | No | Sprotte | 32 |
| 63 | . | F | <45 | . | Depression | Control | Control | 0 | 0 | 0 | 3.5 | No | No | No | No | No | No | No | Sprotte | 22 |
| 64 | . | F | <45 | . | None | Control | Control | 0 | 0 | 0 | 2.4 | No | No | No | No | No | No | Yes | Sprotte | 24 |
| 65 | . | F | <45 | . | None | Control | Control | 0 | 0 | 0 | 3.5 | No | No | No | No | No | No | No | Sprotte | 22 |
| 66 | . | F | <45 | . | None | Control | Control | 0 | 0 | 0 | 2.9 | No | No | No | No | No | No | No | Sprotte | 22 |
| 67 | . | F | >=45 | . | None | Control | Control | 0 | 0 | 0 | 3.7 | No | No | No | No | No | No | No | Sprotte | 22 |
| 68 | . | M | >=45 | . | None | Control | Control | 0 | 0 | 0 | 8.3 | No | No | No | No | Yes | No | No | Quincke | 24 |
| 69 | . | F | <45 | . | None | Control | Control | 0 | 0 | 0 | 4.1 | No | No | No | No | No | No | No | Sprotte | 22 |
| 70 | . | F | <45 | . | None | Control | Control | 0 | 0 | 0 | 4.5 | No | No | No | No | No | No | No | Sprotte | 24 |
| 71 | . | F | <45 | . | None | Control | Control | 0 | 0 | 0 | 5 | No | No | No | No | No | No | No | Sprotte | 24 |
| 72 | . | F | <45 | . | None | Control | Control | 0 | 0 | 0 | 4.9 | No | No | No | No | No | No | Yes | Sprotte | 22 |
| 73 | . | M | <45 | . | None | Control | Control | 0 | 0 | 0 | 4.9 | No | No | No | No | No | No | No | Sprotte | 24 |
| 74 | . | M | <45 | . | None | Control | Control | 0 | 0 | 0 | 4.3 | No | No | No | No | No | No | No | Sprotte | 28 |
| 75 | . | M | <45 | . | None | Control | Control | 0 | 0 | 0 | 7.9* | No | No | No | No | No | No | No | Sprotte | 24 |
| 76 | . | M | <45 | . | None | Control | Control | 0 | 0 | 0 | 2.9 | No | No | No | No | No | No | Yes | Quincke | 32 |
| 77 | . | M | >=45 | . | None | Control | Control | 0 | 0 | 0 | 2.6 | No | No | No | No | Yes | No | No | Quincke | 26 |
| 78 | . | F | >=45 | . | Depression | Control | Control | 0 | 0 | 0 | 3.6 | No | No | No | No | No | No | No | Sprotte | 22 |
| 79 | . | F | >=45 | . | None | Control | Control | 0 | 0 | 0 | 6.4 | No | No | No | No | Yes | No | No | Quincke | 20 |
| 80 | . | F | >=45 | . | None | Control | Control | 0 | 0 | 0 | 6.6 | No | No | No | No | No | No | No | Sprotte | 22 |
| 81 | . | F | >=45 | . | None | Control | Control | 0 | 0 | 0 | 6.9 | No | No | No | No | No | No | No | Quincke | 22 |
| 82 | . | F | >=45 | . | Depression | Control | Control | 0 | 0 | 0 | 3.7 | No | No | No | No | No | No | No | Sprotte | 24 |
| 83 | . | M | <45 | . | None | Control | Control | 0 | 0 | 0 | 2.3 | No | No | No | No | No | No | No | Quincke | 24 |
| 84 | . | F | <45 | . | None | Control | Control | 0 | 0 | 0 | 4.8 | No | No | No | No | No | No | No | Sprotte | 20 |
| 85 | . | F | >=45 | . | None | Control | Control | 0 | 0 | 0 | 3.5 | No | No | No | No | Yes | No | No | Quincke | 28 |
| 86 | . | M | >=45 | . | None | Control | Control | 0 | 0 | 0 | 5.4 | No | No | No | No | No | No | No | Quincke | 22 |
| 87 | . | F | <45 | . | None | Control | Control | 0 | 0 | 0 | 3.9 | No | No | No | No | Yes | No | No | Sprotte | 22 |
| 88 | . | M | <45 | . | None | Control | Control | 0 | 0 | 0 | 4.7 | No | No | No | No | No | No | No | Sprotte | 26 |
| 89 | . | F | >=45 | . | None | Control | Control | 0 | 0 | 0 | 4 | No | No | No | No | No | No | No | Sprotte | 22 |
| 90 | . | F | <45 | . | None | Control | Control | 0 | 0 | 0 | 3.1 | No | No | No | No | No | No | No | Sprotte | 20 |
| 91 | . | M | >=45 | . | None | Control | Control | 0 | 0 | 0 | 9.8 | No | No | No | No | No | No | Yes | Quincke | 24 |
| 92 | . | F | <45 | . | None | Control | Control | 0 | 0 | 0 | 3.8 | No | No | No | No | No | No | Yes | Sprotte | 22 |
| 93 | . | M | >=45 | . | None | Control | Control | 0 | 0 | 0 | 7 | No | No | No | No | Yes | No | No | Quincke | 22 |
| 94 | . | M | <45 | . | None | Control | Control | 0 | 0 | 0 | 10.4* | No | No | No | No | No | No | No | Sprotte | 26 |
| 95 | . | M | <45 | . | None | Control | Control | 0 | 0 | 0 | 7.9* | No | No | No | No | No | No | No | Sprotte | 26 |
| 96 | . | F | <45 | . | None | Control | Control | 0 | 0 | 0 | 3.4 | No | No | No | No | No | No | Yes | Quincke | 20 |
| 97 | . | F | <45 | . | None | Control | Control | 0 | 0 | 0 | 4.8 | No | No | No | No | No | No | No | Sprotte | 28 |
| 98 | . | F | <45 | . | None | Control | Control | 0 | 0 | 0 | 3.7 | No | No | No | No | No | No | No | Sprotte | 24 |
| 99 | . | F | >=45 | . | None | Control | Control | 0 | 0 | 0 | 4.4 | No | No | No | No | No | No | No | Quincke | 26 |
| 100 | . | F | <45 | . | None | Control | Control | 1 | 1 | 0 | 5.8 | No | No | No | No | No | No | No | Sprotte | 22 |
| 101 | . | F | <45 | . | None | Control | Control | 0 | 0 | 0 | 5 | No | No | No | No | No | No | No | Sprotte | 18 |
| 102 | . | M | <45 | . | None | Control | Control | 0 | 0 | 0 | 4.8 | No | No | No | No | No | No | No | Both | 24 |

1. F=Female, M=Male

2. In years

3. MZ=Monozygotic, DZ=Dizygotic

4. 1=Positive microscopic CSF finding, 0=No finding.

5. Positive CSF-result from the first fraction: Category 1=Few structures, 2=several structures, 3=Many structures, 0=No structures.

6. Positive CSF-result from the second fraction: Category 1=Few structures, 2=several structures, 3=Many structures, 0=No structures.

7. Albumin ratio: CSF albumin x 10^3^ ⁄ serum albumin. * indicates blood-CSF barrier dysfunction defined as: albumin ratio >6.8 in individuals 44 years or younger and >10.2 in individuals 45 years or older according to Blennow et. al., 1993

8. Life-time alcohol problem was defined as alcohol abuse or dependence ever

9. Life time anxiety disorder was defined as any anxiety disorder ever

10. Type of needle used at lumbar puncture (LP): Quincke needle: 0.70 x 75 mm in diameter and Sprotte needle: 0.50 x 90 mm.

11. BMI=Body mass index
